# Experimental evidence of Fano resonances in nanomechanical resonators

**DOI:** 10.1038/s41598-017-01147-y

**Published:** 2017-04-21

**Authors:** Stefano Stassi, Alessandro Chiadò, Giuseppe Calafiore, Gianluca Palmara, Stefano Cabrini, Carlo Ricciardi

**Affiliations:** 1 Department of Applied Science and Technology, Politecnico di Torino, Corso Duca degli Abruzzi 24, 10128 Torino, Italy; 2 Molecular Foundry, Lawrence Berkeley National Laboratory, 67 Cyclotron Road, Berkeley, California 94720 United States

## Abstract

Fano resonance refers to an interference between localized and continuum states that was firstly reported for atomic physics and solid-state quantum devices. In recent years, Fano interference gained more and more attention for its importance in metamaterials, nanoscale photonic devices, plasmonic nanoclusters and surface-enhanced Raman scattering (SERS). Despite such interest in nano-optics, no experimental evidence of Fano interference was reported up to now for purely nanomechanical resonators, even if classical mechanical analogies were referred from a theoretical point of view. Here we demonstrate for the first time that harmonic nanomechanical resonators with relatively high quality factors, such as cantilevers vibrating in vacuum, can show characteristic Fano asymmetric curves when coupled in arrays. The reported findings open new perspectives in fundamental aspects of classical nanomechanical resonators and pave the way to a new generation of chemical and biological nanoresonator sensors with higher parallelization capability.

## Introduction

Resonance is a physical phenomenon that can be found in a variety of classical and quantum systems. Independently of the complexity of such systems, the spectral dependence is generally represented by symmetric Lorentzian curves. In 1961, the Italian-American physicist Ugo Fano showed that the quantum interference between a continuum of states (the inelastic scattering of electrons by helium atoms) with discrete states (the resonant scattering via autoionization) results in a new resonance behavior, characterized by an asymmetric shape^1^. In the following years, the so-called Fano resonance showed its universal behavior in physics, because, as a manifestation of interference between a localized wave with propagating states, it is independent on the matter. Out of atomic physics, Fano resonances were found in solid-state quantum systems (such as nanowires and quantum dots^2, 3^), as well as in nanoscale photonic devices and metamaterials^4, 5^. Thanks to its steep dispersion, the Fano resonance was successfully exploited to enhance nonlinear optical processes and to engineer the sensing capability of platforms based on plasmonic nanoclusters^6–8^ and SERS^9, 10^.

Parallel to plasmonics and metamaterials, unprecedented progresses have been made in the very recent years in the field of nanomechanical systems for ultrasensitive measurements of force^11^ and displacement^12^, for biosensing^13, 14^ and for molecule/nanoparticle localization^15, 16^. Fano-like resonances were observed in cavity optomechanical systems, where the motion of a nanomechanical oscillator is coupled with an optical signal^17, 18^. In these systems, a laser is coupled to a mechanical resonator, so that its mechanical stiffness (and therefore its frequency response) can be optically tuned. When the optical signal is not coupled to the cavity system, the mechanical resonator maintains its independent resonance frequency. Indeed, the interference that generates Fano resonance when classical resonators are coupled in a purely mechanical way was postulated just theoretically^2, 9^, without any experimental evidence reported up to now. When considering harmonic oscillators with high quality factors such as micro and nanocantilever arrays, an elastic coupling is typically employed to induce vibration localization, thus improving mass sensitivity^19–22^. A suspended overhang is introduced to connect all the identical size N cantilevers of an array, thus generating entangled eigenstates of the whole structure and splitting the resonance frequency of a single cantilever into N frequencies^23^. Femtogram mass resolution was later reached in elastically coupled nanocantilevers array by Tamayo *et al*., exploiting their stochastic and deterministic resonance responses^24^.

In this work, it is shown that the vibration amplitude spectrum of a single nanomechanical resonator in an array can show asymmetric peaks characteristic of Fano resonances in correspondence of the resonance frequencies of the other oscillators of the array. For completeness, Fano resonances have been also studied in fabricated and commercial microcantilevers arrays and a theoretical model to describe the phenomenon is proposed. Furthermore, a cantilever-based immunoassay was performed to proof the peculiar sensitivity of Fano resonances to small physical perturbations in a coupled system.

## Results

### Fano resonances in nanomechanical resonators

The vibration analysis was performed on two different nanomechanical oscillator arrays (see Methods) for first flexural modes, both of them composed of silicon nitrate cantilevers with a thickness of 100 nm. The smaller have nominally length and width of 10 μm and 1 μm, respectively, while the bigger 20 μm and 2 μm. Due to manufacturing tolerances, cantilevers with nominally identical dimensions exhibit slightly different resonance frequencies. In addition, the elastic coupling between vibrating structures is here designed to be relatively weak, because the cantilevers are directly connected to the bulk silicon without any suspended overhang at the base. As a consequence, each cantilever resonance should be well identified by a Lorentzian curve and no entangled states are envisioned. A laser Doppler vibrometer (LDV) system was employed to enable highly precise measurements with sub-picometer resolution and increased signal to noise ratio with respect to optical lever detection^25, 26^. The measurements were performed exciting the resonator arrays with a piezoelectric disk, while the vibration of the cantilevers was evaluated at different frequencies with the LDV system, focusing the laser spot on the tip of the resonator.

**Figure 1:**
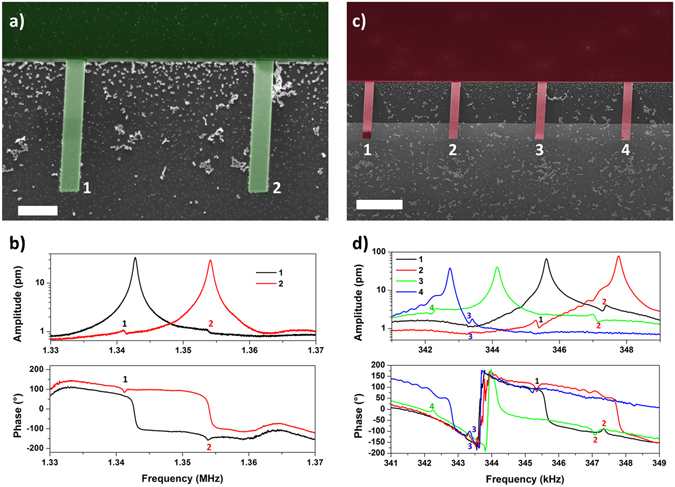
Fano resonances in nanomechanical resonators. (**a**) SEM image of the array of two smaller nanocantilevers. The scale bar corresponds to 2 µm. (**b**) Vibration spectra and phase signals of the cantilevers of array presented in (**a**). The numbers underline the nanocantilevers relative to Fano asymmetric peaks. (**c**) SEM image of the array of four bigger nanocantilevers. The scale bar corresponds to 5 µm. (**d**) Vibration spectra and phase signals of the cantilevers of array presented in (**c**). Please note that initial phase is arbitrary, but phase values should be varying in the range −180°: 180°.

Figure 1 shows the amplitude and phase spectra of the two different nanoresonator arrays where Fano resonances are clearly visible. For each nanocantilever, the Lorentzian peak is clearly visible, while, as expected, no elastic entangled modes are present. Instead, asymmetric peaks appear exactly in correspondence of the resonance frequencies of the other cantilevers. Indeed, Fano resonances are created from the interference between the broad tails of the Lorentzian resonance spectrum of the examined cantilever (the continuum state), with the narrow resonance peaks of the other cantilevers in the array (localized states). The cantilever spectra were plotted in logarithmic scale to enhance the contribution from the Fano resonance peaks, which are characterized by very small amplitudes, even 40 dB below the Lorentzian resonance peaks. As expected, Fano resonances appear also in the phase signal, where, in addition to the phase inversion typical of the Lorentzian resonance mode, different asymmetric peaks are shown in correspondence of the phase inversions of the other cantilevers. In the array composed by two smaller nanoresonators (10 µm × 1 µm × 100 nm, Fig. 1a,b), only one Fano peak appears which is related to the cantilever not under test. Instead in the array with 4 bigger nanoresonators (20 µm × 2 µm × 100 nm, Fig. 1c,d), the response is more complex. Since the amplitude of the Fano peaks in nanocantilevers array is very small and the damping of the signal increases moving far in space from the measured oscillator, only asymmetric Fano peaks related to the first neighbor cantilevers are visible. Therefore oscillators 1 and 4 reported only one Fano peak each and oscillators 2 and 3 two. Actually in these nanoresonators arrays the Fano peaks are slightly moved to lower frequency values with respect of the corresponding cantilevers. This phenomenon is probably connected to the slowing of phonon mobility in the nanometric layer that composes the resonators. In fact, it is well known that acoustic phonons suffer a reduction of their phase and group velocities in nanometric system due to nanoscale confinement, surface roughness scattering and acoustic mismatch between Si_3_N_4_ layer and bulk silicon^27, 28^.

### Measurements on microcantilever arrays

**Figure 2:**
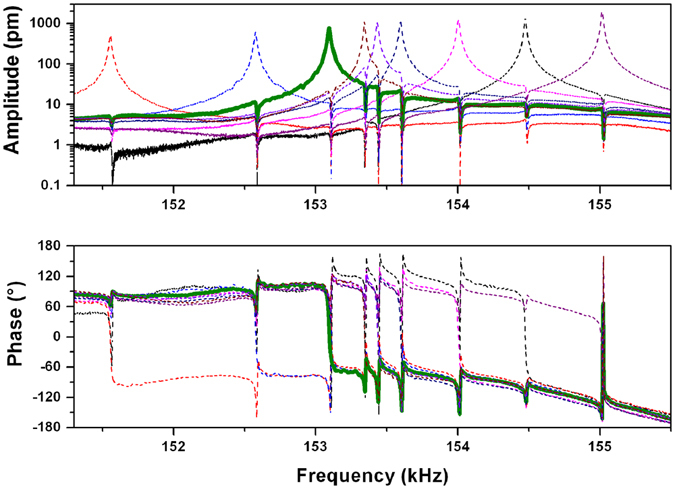
Fano resonances in amplitude and phase spectra. Vibration spectra and phase signals of all the cantilevers of the MC_9 array, centered around the second resonance mode. The spectrum of the central cantilever is represented with a thick continuous line, while all the others by dotted lines.

Fano resonance peaks are much easily observable in microcantilever arrays, where signal amplitude is higher with respect to nanoresonators and thus the coupling is visible not only for the first neighbor oscillators. Figure 2 shows the amplitude and phase signal of the second resonance mode of the central structure of the array composed by 9 cantilevers (MC_9). The Lorentzian amplitude peak is clearly visible and centered around 153.097 kHz and all the eight asymmetric peaks appear exactly in correspondence of the resonance frequencies of the other cantilevers (Lorentzian dotted curves in Fig. 2). As often found in the literature, Fano peaks exhibit a more pronounced reduction of the amplitude respect to a perfectly asymmetric shape, probably because the Lorentzian tail representing the continuum state is characterized by a slowly varying amplitude, i.e. not exactly constant (as theoretically expected). The same behavior is observable for phase signal. In microcantilever arrays Fano peaks do not present any shift to lower frequency, like for nanoresonators, since nanoscale confinement, surface and interface effects become negligible (all the microcantilevers under analysis have a thickness of 7 µm).

**Figure 3:**
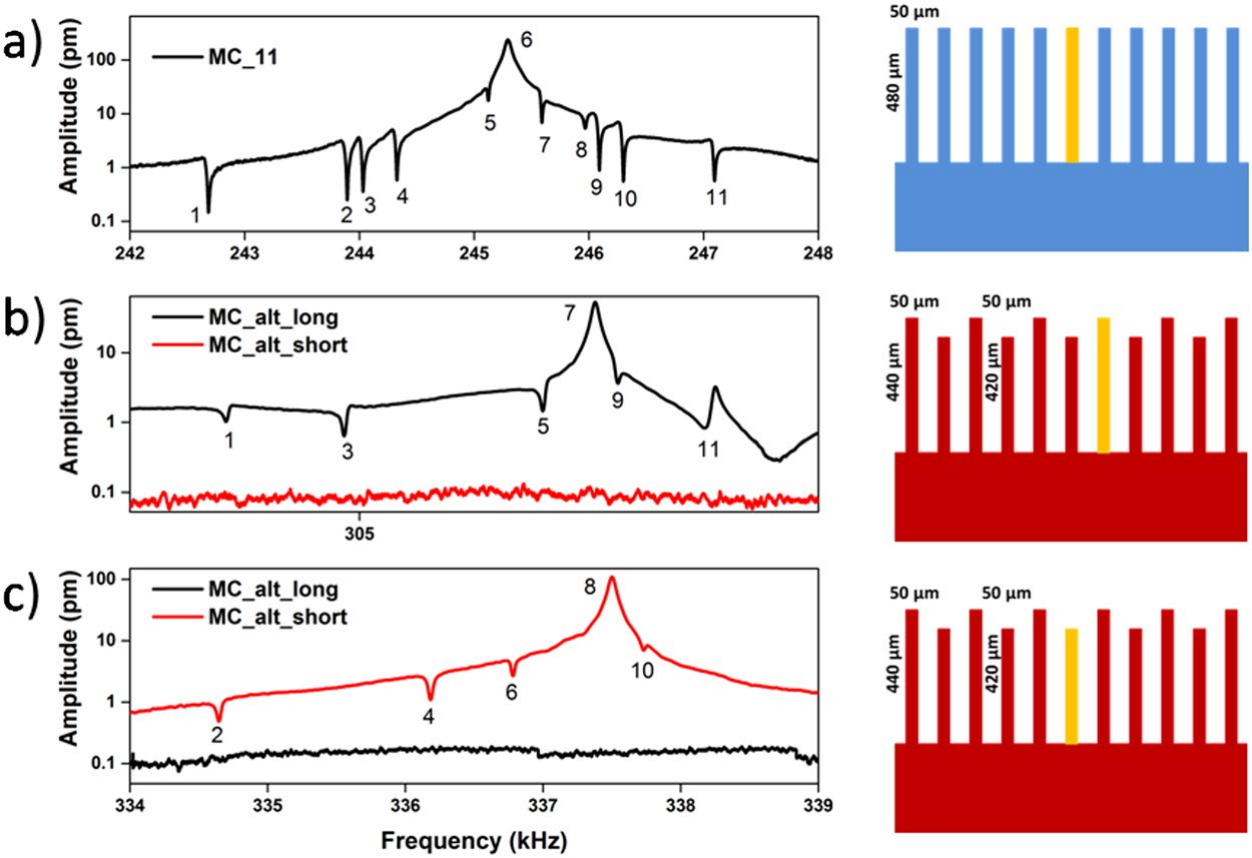
Generalization of Fano resonances in mechanical oscillators. (**a**) Vibration spectra of the central cantilever of MC_11 array, centered around the second resonance mode. (**b**) and (**c**) Vibration spectra of the central cantilevers of MC_alt array, centered around the second resonance mode of the long and short cantilevers, respectively. The numbers are not referred to the cantilever position in the array; they are just a numerical counter to label the presence of all the expected peaks. Aside of each graph a scheme of the cantilever array is reported: the structure under analysis is highlighted.

Other microcantilever arrays with different geometries were also tested to show the generalization of our finding. Figure 3a shows the second resonance mode of the central structure of an array composed by 11 nominally identical cantilevers (MC_11). As expected, ten Fano asymmetric peaks appear exactly in correspondence of the resonance frequencies of the other cantilevers. On the other hand, if an array composed by alternate length structures (MC_alt) is used, the experimental spectra are interestingly different. Figure 3b,c shows the second resonance mode of the central structures of a MC_alt array. Owing to the large difference in resonance frequency (*f*_*r*_ = 306.540 kHz for the long ones, *f*_*r*_ = 337.500 kHz for the short ones), Fano resonances are experimentally measurable just for the cantilevers of nominal identical dimensions (five peaks in Fig. 3b, four in Fig. 3c), while the other interferences are covered by signal noise. Thus, it is clear that the intensity of the Fano peaks here depends more on the separation in the resonance curves (frequency and width) of the oscillators, rather than on the physical distance among them. Fano resonances were also measured in commercial cantilever arrays fabricated by IBM, even if they were fabricated with a technological expedient commonly used to avoid the so-called “cross-talk” effect between oscillators with similar resonance response (Figure S2 in S.I.).

**Figure 4:**
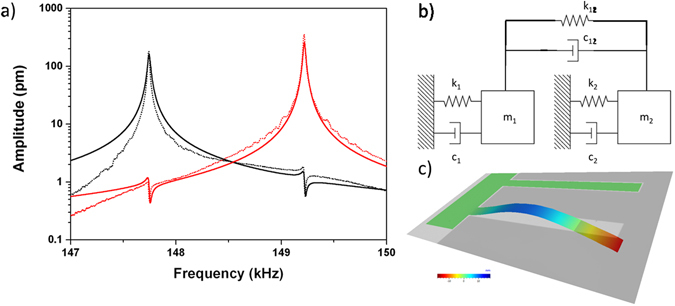
Simulation of Fano resonances in coupled resonators. (**a**) Comparison of the vibration spectra of the two cantilevers with physical dimension of MC_9 resonators in a weakly coupled array. Solid lines represent the numerical calculation, while the dotted lines are the experimental data. (**b**) Schematic model of the coupled cantilever array. (**c**) 3-D visualization of the device experimental amplitude at the second resonance frequency of the first cantilever.

### Theoretical model of Fano resonances in mechanical resonators

A numerical modeling of the experimental data is proposed (Fig. 4), generalizing the classical analogy reported by Joe *et al*.^9^ to an array in which both the oscillators (not just one) are subjected to the same external harmonic force (as in our experimental set-up, where the whole array is glued to the piezoactuator). Each cantilever *n* (with *n* = *1*,*2*) was modeled by a damped harmonic oscillator constituted by active mass *m*_*n*_, structural dissipation *c*_*n*_ and bending stiffness *k*_*n*_. The coupling element was modeled with a weak spring constant *k*_*12*_, so that the two eigenmodes were negligibly shifted from the solutions of independent oscillators and with a damping element *c*_*12*_ (Fig. 4b).

The dynamics of the above described resonating structure subject to external periodic driving force *F·e* ^*iωt*^ is governed by the following differential equation system:

1 $$\begin{matrix}{\ddot{x}}_{1}+{\gamma }_{1}{\dot{x}}_{1}+{{\omega }_{1}}^{2}{x}_{1}+{\upsilon }_{12}({x}_{1}-{x}_{2})+{\gamma }_{12}({\dot{x}}_{1}-{\dot{x}}_{2}) & = & F\cdot {e}^{i\omega t}\\ {\ddot{x}}_{2}+{\gamma }_{2}{\dot{x}}_{2}+{{\omega }_{2}}^{2}{x}_{2}+{\upsilon }_{21}({x}_{2}-{x}_{1})+{\gamma }_{21}({\dot{x}}_{2}-{\dot{x}}_{1}) & = & F\cdot {e}^{i\omega t}\end{matrix}$$

where ${\omega }_{n}=\sqrt{{k}_{n}/{m}_{n}}$ are the natural frequencies of the single resonators, ${\gamma }_{n}={c}_{n}/{m}_{n}$ the frictional parameter and ${\upsilon }_{12}={\upsilon }_{21}={k}_{12}/{m}_{n}$ and ${\gamma }_{12}={\gamma }_{21}={c}_{12}/{m}_{n}\,\,$the elastic and damping coupling between the cantilevers.

Since the forced response of a damped system has the same frequency as the driving force, but with different amplitude and phase, the solutions of the system are assumed in the form:

2 $${x}_{1,2}={X}_{1,2}(\omega ){e}^{i\omega t}$$

where the complex amplitude can be written as:

3 $$\begin{matrix}{a}_{1}(\omega )=\\ \quad \frac{({{\rm{\omega }}}_{2}^{2}-{{\rm{\omega }}}^{2}+i{\gamma }_{2}\omega +2{\upsilon }_{12}+2i{\gamma }_{12}\omega )}{({{\rm{\omega }}}_{1}^{2}-{{\rm{\omega }}}^{2}+i{\gamma }_{1}\omega )({{\rm{\omega }}}_{2}^{2}-{{\rm{\omega }}}^{2}+i{\gamma }_{2}\omega )+{\upsilon }_{12}({{\rm{\omega }}}_{1}^{2}-{{\rm{\omega }}}^{2}+i{\gamma }_{1}\omega )+{\upsilon }_{12}({{\rm{\omega }}}_{2}^{2}-{{\rm{\omega }}}^{2}+i{\gamma }_{2}\omega )+i{\gamma }_{12}\omega ({{\rm{\omega }}}_{1}^{2}-{{\rm{\omega }}}^{2}+i{\gamma }_{1}\omega )+i{\gamma }_{12}\omega ({{\rm{\omega }}}_{2}^{2}-{{\rm{\omega }}}^{2}+i{\gamma }_{2}\omega )}F\\ {a}_{2}(\omega )=\\ \quad \frac{({{\rm{\omega }}}_{1}^{2}-{{\rm{\omega }}}^{2}+i{\gamma }_{1}\omega +2{\upsilon }_{12}+2i{\gamma }_{12}\omega )}{({{\rm{\omega }}}_{1}^{2}-{{\rm{\omega }}}^{2}+i{\gamma }_{1}\omega )({{\rm{\omega }}}_{2}^{2}-{{\rm{\omega }}}^{2}+i{\gamma }_{2}\omega )+{\upsilon }_{12}({{\rm{\omega }}}_{1}^{2}-{{\rm{\omega }}}^{2}+i{\gamma }_{1}\omega )+{\upsilon }_{12}({{\rm{\omega }}}_{2}^{2}-{{\rm{\omega }}}^{2}+i{\gamma }_{2}\omega )+i{\gamma }_{12}\omega ({{\rm{\omega }}}_{1}^{2}-{{\rm{\omega }}}^{2}+i{\gamma }_{1}\omega )+i{\gamma }_{12}\omega ({{\rm{\omega }}}_{2}^{2}-{{\rm{\omega }}}^{2}+i{\gamma }_{2}\omega )}F\end{matrix}$$

The real amplitude of the cantilever is defined by the modulus $|{a}_{n}(\omega )|$ and phase as:

4 $${a}_{n}(\omega )=|{a}_{n}(\omega )|{e}^{i{\phi }_{n}(\omega )}$$

As shown in Fig. 4a, the experimental curves of a vibrating array composed of two weakly coupled cantilevers, with the same physical dimensions of the cantilevers of the MC_9 array, exhibit two characteristic asymmetric Fano resonances that are in very good agreement with the numerical solutions obtained from the modulus of Equation (3). The elastic and damping coupling constant used in the numerical simulation were estimated as ${\upsilon }_{12}={10}^{8}\,\,{s}^{-2}$ and ${\gamma }_{12}=30\,\,{s}^{-1}$ (detailed parameters are reported in S.I.). A good agreement between experimental and simulated data has been obtained also for the other resonator geometries, i.e. MC_11 and MC_alt (data shown in the S.I.). The model and the numerical computation applied here for simplicity to the second flexural mode of two weakly coupled cantilevers can be straightforwardly extended to larger arrays, with different resonators and vibrational modes.

### Parallelization of cantilever resonance analysis

**Figure 5:**
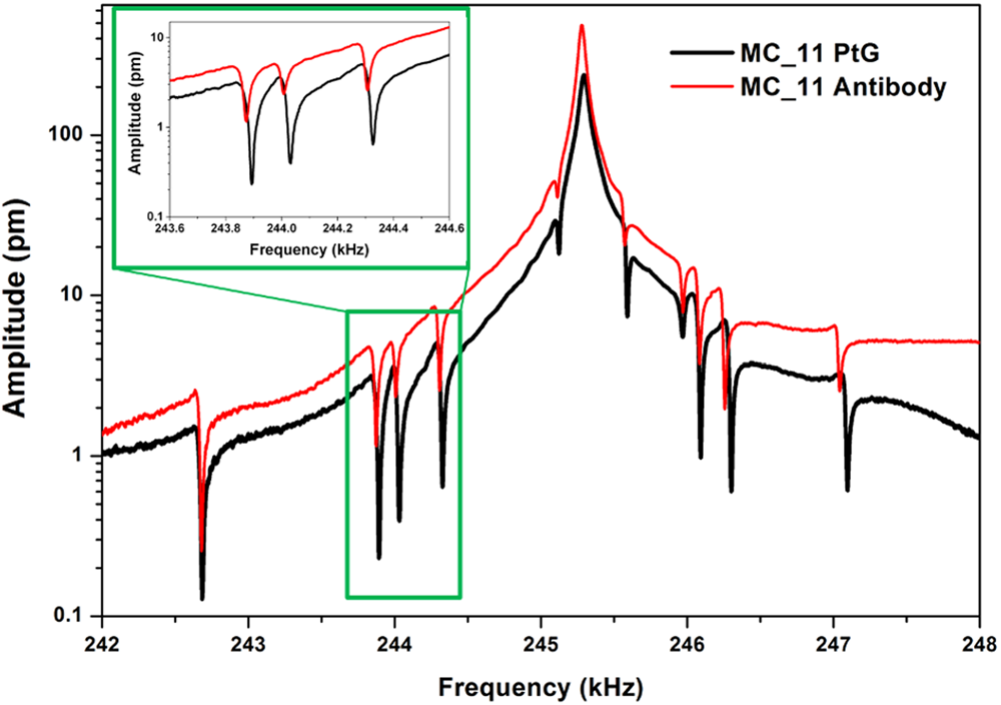
Parallelization of cantilever resonance analysis. Vibration spectra of the central cantilever of MC_11 array centered around the second resonance mode measured after the incubation with Protein G (black line) and after the antibody binding (red line).

Beyond the physical discover, Fano interference in classical mechanics could be exploited to considerably improve the application of micro and nano-resonators to chemical and biological sensors. To demonstrate such a potentiality, a biochemical assay was performed. The vibrational spectrum of the central cantilever of the array was acquired on the bare chip and after every protocol step: the functionalization with APTES/SA, the incubation with Protein G and the antibody binding. As shown in Fig. 5, both Lorentzian and Fano resonances experienced coherent negative frequency shifts, due to the antibody mass loading homogenously distributed on all the microcantilevers. Thus, the here proposed Fano resonance analysis enables the possibility of evaluating the mass adsorbed by all the resonant structures of an array with a single fast measurement, strongly decreasing the analysis time and cost of such bioassays. Since the physical distance among the different structures is not much affecting the appearance of Fano resonances (as previously demonstrated for the different arrays), a high parallelization capability for the proposed method is envisioned. Furthermore, similarly to what happened very recently for nano-optical sensing platforms^5–10^, the steep dispersion of the Fano resonance could be exploited to enhance the assay sensitivity. As an example, the quality factor of Fano peaks in our devices is typically ten times higher than correspondent Lorentzian peaks. The sensitivity as micro/nano-balance can be evaluated as the minimum detectable mass *Δm*, that is typically expressed as *Δm∝m*/*Q*, where m is the mass of the oscillator and Q its quality factor^29^. Thus, in the resonator arrays under study, the minimum detectable mass *Δm* of each cantilever decreases from 40 pg to around 5 pg, thanks to an average ten-fold increase in Q factor of Fano peaks respect to Lorentzian ones.

## Conclusion

In conclusion, we have demonstrated that Fano resonances can be experimentally observed in purely mechanical systems constituted by classical resonators with similar dimensions, like nano and microcantilever arrays. Different geometries were tested and a theoretical model based on both weak elastic and damped coupling well supported the experimental results. Beyond the physical discover, our findings open new perspective in exploiting the peculiar properties of Fano resonances, like delocalization and steep dispersion, to develop a new generation of nanomechanical sensors with higher parallelization capability and enhanced mass sensitivity. As a first proof, the minimum detectable mass of each cantilever was found to decrease of roughly one order of magnitude if Fano resonances are employed, instead of their correspondent Lorentzian peaks.

## Methods

### Microcantilever fabrication and functionalization

For the fabrication of the nanocantilevers, a 150-nm thick layer of ZEP520A was spin coated on a stack of 100 nm Si_3_N_4_ on silicon. A Vistec VB300 electron beam lithography system was used to define the shape of the cantilevers in the ZEP layer. The pattern was transferred into Si_3_N_4_ using reactive ion etching and a mixture of CHF_3_ and 4% O_2_, with 50 W of RF power, a pressure of 55 mT and an etching time of 10 minutes. Cantilevers were finally released by undercutting the silicon in an aqueous solution of 33%wt KOH at 160 °C for 10 minutes. Nanocantilevers were prepared with two different geometries. The smaller have nominally length and width of 10 μm and 1 μm, respectively, while the bigger 20 μm and 2 μm. Both nano-oscillator geometries have thickness of 100 nm.

Tree silicon microcantilever arrays with different dimensions were used in this work. The first chip consisted of 9 cantilevers (MC_9) with nominal length and width respectively of 590 μm and 70 μm the second one of 11 cantilevers (MC_11) with length and width of 480 μm and 50 μm, and the last array was of 11 cantilevers (MC_alt) with width of 50 μm and two different alternate length, six cantilevers of 440 μm and five of 420 μm. All the cantilevers had nominal thickness of 7 μm. They were fabricated using a combination of surface and bulk micromachining techniques following the protocol described in details elsewhere^30, 31^. Briefly, starting from a silicon-on-insulator wafer an opening from the back was fabricated with a KOH etching, while the microcantilever pattern was transferred on the wafer with a reactive ion etching step (RIE). Commercial microcantilever chip with different length was acquired from Concentris (Type CLA-500-070-04V2, Concentris Gmbh, Switzerland).

Cantilever resonance frequencies were monitored with a high precision laser Doppler vibrometer system (MSA-500, Polytec Gmbh) after each step. The measurement system was coupled to a vacuum chamber containing a piezoelectric disk actuator on which microcantilever arrays were mounted with an adhesive tape. For the evaluation of the cantilever resonance frequencies, the measurement chamber was evacuated by a membrane and a turbomolecular pumps (MINI-Task System, Varian Inc. Vacuum Technologies) up to a vacuum level of 2 * 10^−7^ mbar.

### Cantilever functionalization and bioassay protocol

Sulphuric acid (95–98% w/w), hydrogen peroxide (30% w/w), 3-aminopropyltriethoxysilane (APTES, anhydrous, 99%), sodium chloride (≥99.5%), triethylamine, tetrahydrofuran, ethanol (99.9%), %, toluene (anhydrous; 99.8%), 1-ethyl-3-(3-dimethylaminopropyl)carbodiimide hydrochloride (EDC) (99.0%), N-hydroxysulfosuccinimide sodium salt (NHS) (98.0%), tween 20™, Phosphate Buffered Saline (PBS) and 4-morpholineethanesulfonic acid (MES, 99.5%) were purchased from Sigma-Aldrich (Milan, IT). Recombinant Protein G was from Thermo Fisher Scientific (Illkirk, FR), whereas goat anti-mouse IgG, horseradish peroxidase conjugated (Ab-HRP) was from Merk-Millipore (Milan, IT). All water solutions were prepared with Milli-Q™ water (Merk-Millipore, Milan, IT).

MC arrays were immersed in piranha solution (75% H_2_SO_4_, 25% H_2_O_2_ v/v) for 15 min and rinsed three times in deionized water in order to remove organic contaminants. Afterwards, they were silanized following the procedure reported in detail by Chiadò *et al*.^32^. Briefly, the chips were immersed for 10 min at 70 °C in 1% v/v APTES in toluene under anhydrous conditions, then rinsed three times in toluene and dried under a nitrogen stream. Subsequently, the cantilevers were incubated at room temperature in 5 mg/mL succinic anhydride in tetrahydrofuran supplemented with 5% (v/v) triethylamine for 2 h, rinsed three times in THF, ethanol and deionized water and finally dried under a stream of N_2_. Afterwards, the resonance frequencies of the functionalized MC arrays were recorded. The carboxylic groups on the surface of the chips were then activated by means of a EDC/NHS protocol. In particular, the arrays were equilibrated in MES buffer (100 mM MES, 0.9% NaCl, pH 4.7) for 15 min and subsequently incubated in a mixture of EDC (4 mM) and NHS (10 mM) in MES buffer for 15 min, as previously reported^32^. Afterwards, the MC arrays were rinsed three times in PBS and incubated overnight in a Protein G solution (0.05 mg/mL in PBS) at 4 °C, washed thrice in PBS-tween 20™ 0,05% v/v, rinsed three times in deionized water and dried in a nitrogen stream. The resonance frequencies of the MC arrays were recorded again. Cantilever were then incubated in 2 μg/mL Ab-HRP solution for 1 h, at room temperature, then washed thrice in PBS-tween 20™ 0,05% v/v, three times in deionized water and dried in a nitrogen stream. More details on the previously optimized procedures for protein immobilization and detection by resonating cantilevers are reported elsewhere^33, 34^.

## Electronic supplementary material

Supplementary Material
