# Supplementary material for: Experimental evidence of Fano resonances in nanomechanical resonators
Source: Sci Rep. 2017 Apr 21;7:1065. doi: 10.1038/s41598-017-01147-y (PMC5430710; doi:10.1038/s41598-017-01147-y)
Supplement: Supplementary file 1 — Supplementary Material [file 41598_2017_1147_MOESM1_ESM.doc]

**SUPPLEMENTAL MATERIAL**

Experimental evidence of Fano resonances in nanomechanical resonators

**Stefano Stassi^1^,*, Alessandro Chiadò^1^, Giuseppe Calafiore^2^, Gianluca Palmara^1^, Stefano Cabrini^2^, and Carlo Ricciardi^1^**

^1^Department of Applied Science and Technology, Politecnico di Torino, Corso Duca degli Abruzzi 24, 10128 Torino, Italy
^2^Molecular Foundry, Lawrence Berkeley National Laboratory, Berkeley, California 94720, United States

*stefano.stassi@polito.it

**LORENTZIAN FIT OF OSCILLATOR RESONANCE FREQUENCY**


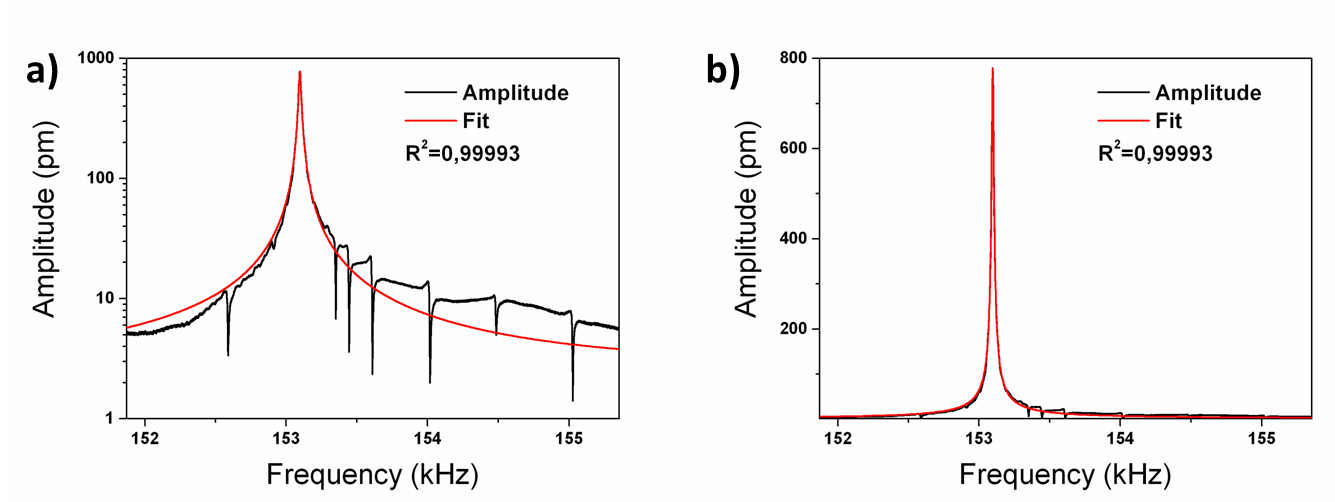


**Figure S1.** Fit with a Lorentzian curve of the resonance frequency of the central cantilever of MC_9 (see green curve in Fig.2 of the article) in a) logarithmic and b) linear scale

Here we show that the resonance curves of our cantilevers have a typical Lorentzian behavior, as observable in the fit presented in the Fig. S1 (in logarithmic and linear scale). As example we fit the green curve of Figure 2 and the R^2^ resulted 0.99993. Even if the R^2^ is very close to 1, in the logarithmic plot is possible to notice that far from the resonance, the background is slightly increasing with the frequency due to instrument noise, even if in the picometer range.

**FANO RESONANCES IN COMMERCIAL CANTILEVER ARRAYS**

We characterized commercial microcantilever arrays fabricated by IBM, where the bulk silicon was extended over the base of the cantilevers (as shown in the image and sketch in Fig.S2) to better separate the eight individual cantilevers, with four different lengths reproduced twice. Such a technological expedient is commonly used to avoid the so-called "cross-talk" effect between oscillators with similar resonance response. As shown in Fig.S2, every couple of homologous resonators shows two Lorentzian curves with the two correspondent Fano asymmetric peaks, confirming the generality of our findings.


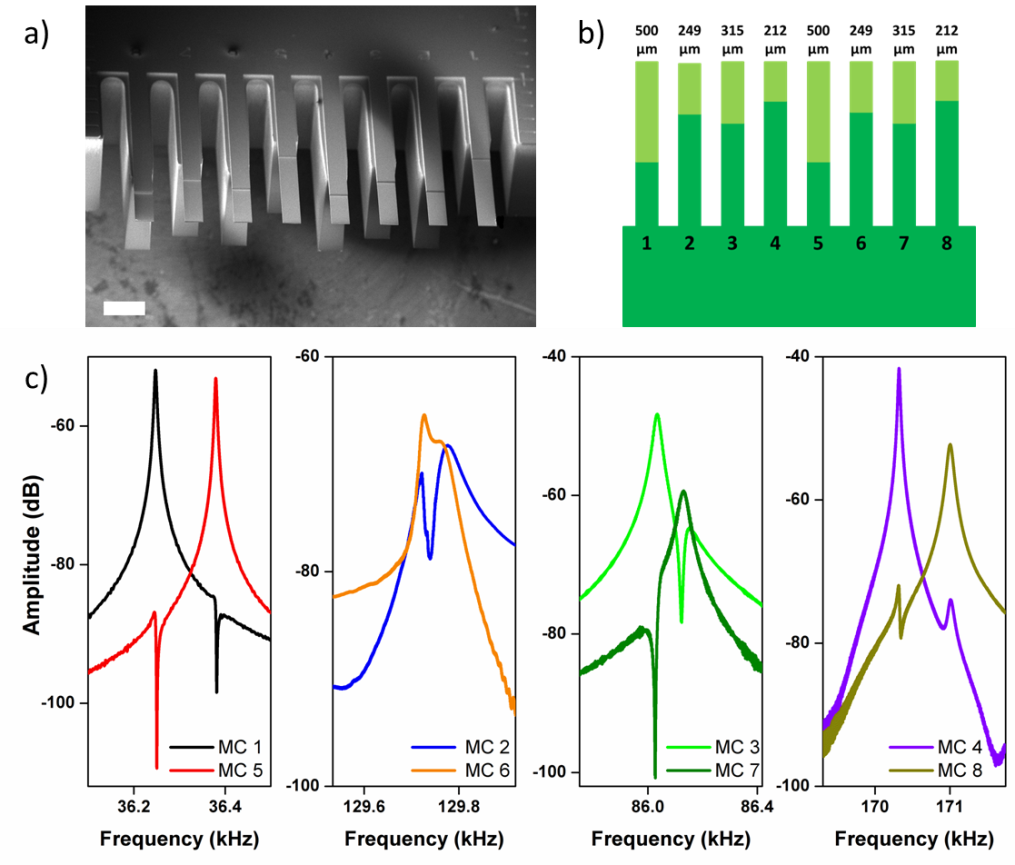


**Figure S2** Fano resonances in commercial arrays a) Scanning Electron Microscopy image and b) schematic of the IBM cantilever array. C) Vibration spectra of the cantilevers of the Concentris array centered around the first resonance mode showing the damping coupling effect.

**NUMERIC SIMULATION PARAMETERS**

The numeric simulation has been performed with a Matlab script. Here below are reported the numeric parameters used in the two weakly coupled cantilever model. Mass and frequency of both resonators were experimentally measured, while the other parameters were optimized to increase the accuracy of the fit.

| **PARAMETER** | **SYMBOL** | **VALUE** |
| --- | --- | --- |
| Cantilever mass | *m_1_=m_2_* | 639 ng |
| Force over mass | *F* | 170 N kg^-1^ |
| Resonance frequency first cantilever | *ω_1_* | 2π*147735 Hz |
| Resonance frequency second cantilever | *ω_2_* | 2π*149210 Hz |
| Frictional parameter first cantilever | *γ_1_* | 210 s^-1^ |
| Frictional parameter second cantilever | *γ_2_* | 200 s^-1^ |
| Elastic coupling parameter | *υ_12_= υ_21_* | 10^8^ s^-2^ |
| Damping coupling parameter | *γ_12_= γ_21_* | 30 s^-1^ |

**NUMERIC SIMULATION FOR OTHER RESONATOR GEOMETRIES**

The numeric simulation has been carried out also for the other types of resonator geometries: MC_11, as well as long and short cantilevers of MC_alt. A good agreement between experimental and simulated data has been obtained for all the resonators, as shown in the figures S3, S4 and S5.

**
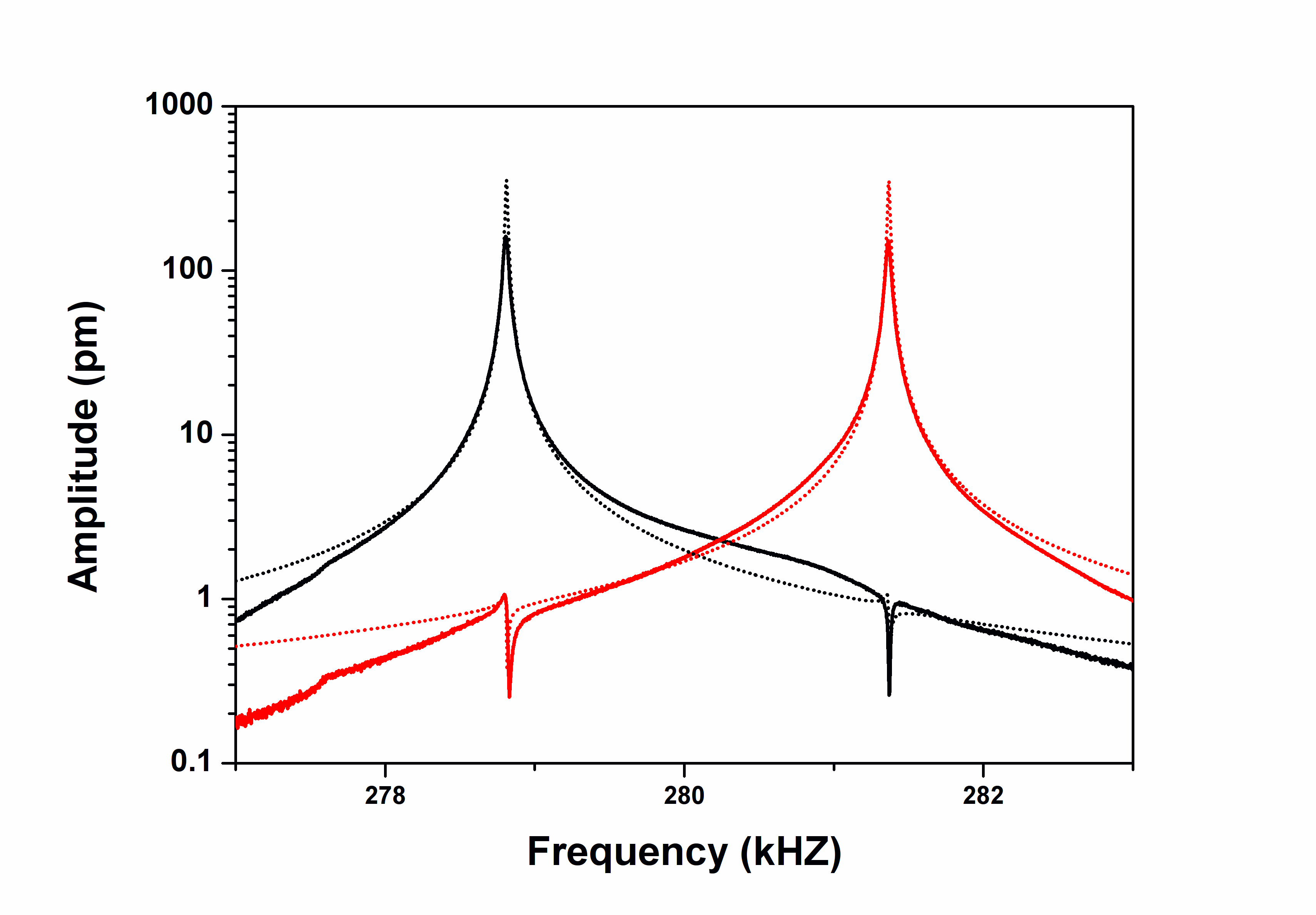
**

| **PARAMETER** | **VALUE** |
| --- | --- |
| *m_1_=m_2_* | 391 ng |
| *F* | 170 N kg^-1^ |
| *ω_1_* | 2π*278806 Hz |
| *ω_2_* | 2π*281362 Hz |
| *γ_1_* | 150 s^-1^ |
| *γ_2_* | 150 s^-1^ |
| *υ_12_= υ_21_* | 10^8^ s^-2^ |
| *γ_12_= γ_21_* | 50 s^-1^ |

**Figure S3.** Comparison of the experimental and simulated vibration spectra of the two cantilevers in a weakly coupled array with physical dimension of MC_11 resonators. Solid lines represent the numerical calculation, while the dotted lines are the experimental data.

**
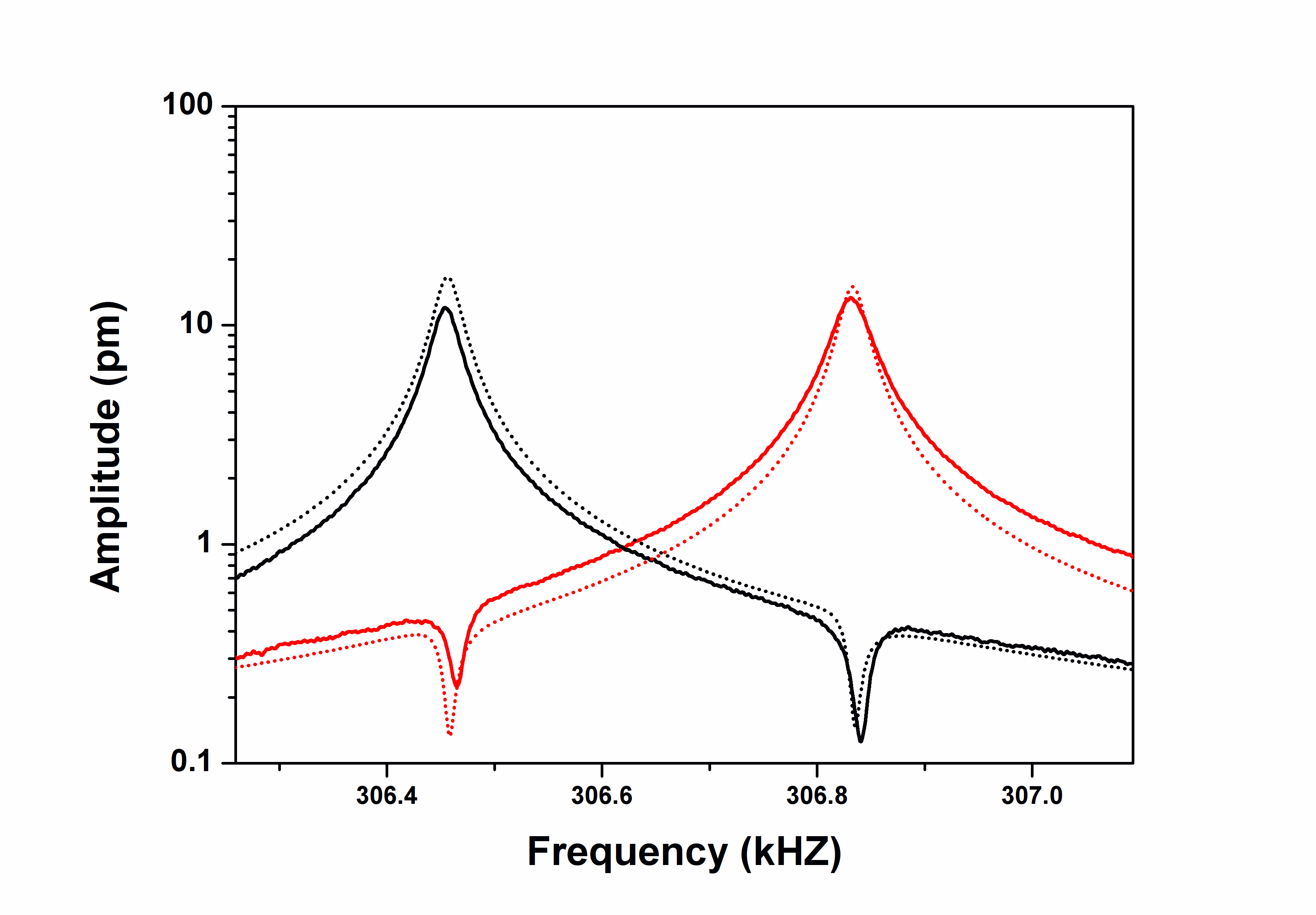
**

| **SYMBOL** | **VALUE** |
| --- | --- |
| *m_1_=m_2_* | 359 ng |
| *F* | 80 N kg^-1^ |
| *ω_1_* | 2π*306456 Hz |
| *ω_2_* | 2π*306835 Hz |
| *γ_1_* | 350 s^-1^ |
| *γ_2_* | 360 s^-1^ |
| *υ_12_= υ_21_* | 5*10^7^ s^-2^ |
| *γ_12_= γ_21_* | 160 s^-1^ |

**Figure S4.** Comparison of the experimental and simulated vibration spectra of the two cantilevers in a weakly coupled array with physical dimension of MC_alt long resonators. Solid lines represent the numerical calculation, while the dotted lines are the experimental data.

**
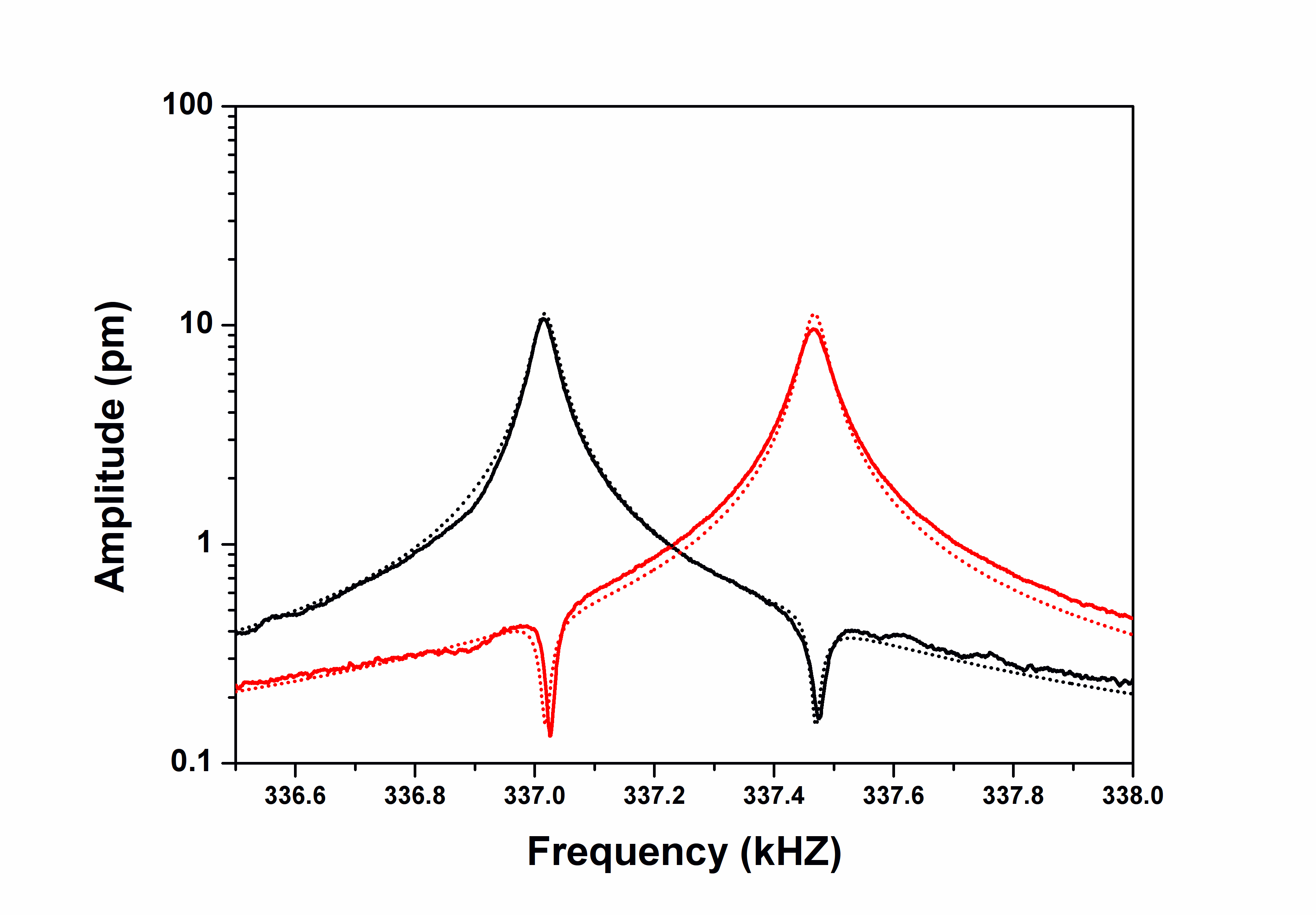
**

| **SYMBOL** | **VALUE** |
| --- | --- |
| *m_1_=m_2_* | 343 ng |
| *F* | 80 N kg^-1^ |
| *ω_1_* | 2π*337014 Hz |
| *ω_2_* | 2π*337465 Hz |
| *γ_1_* | 400 s^-1^ |
| *γ_2_* | 400 s^-1^ |
| *υ_12_= υ_21_* | 5*10^7^ s^-2^ |
| *γ_12_= γ_21_* | 160 s^-1^ |

**Figure S5.** Comparison of the experimental and simulated vibration spectra of the two cantilevers in a weakly coupled array with physical dimension of MC_alt short resonators. Solid lines represent the numerical calculation, while the dotted lines are the experimental data.
